# Non-Thermal Plasma and Ho: YAG Laser Surface Conditioning: Effective Superior Alternatives to Sandblasting for 3Y-TZP Zirconia-Composite Repair Bonding

**DOI:** 10.12669/pjms.42.6.15464

**Published:** 2026-06

**Authors:** Amer Alanazi, Mustafa Naseem, Micheal Pfitzner, Haifa Arsalan

**Affiliations:** 1 Amer M. Alanazi, Pharmaceutical Biotechnology Laboratory, Department of Pharmaceutical Chemistry, College of Pharmacy, King Saud University, Riyadh 11451, Saudi Arabia; 2 Mustafa Naseem, Department of Community Preventive Dentistry, Dow International Dental College, Dow University of Health Sciences, Karachi, Pakistan; 3 Micheal Pfitzner, Department of Restorative Dentistry, Cardiff University, UK; 4 Haifa Arsalan, Dow University of Health Sciences, Dr. Ishrat-ul-Ebad Khan, Institute of Oral Health Sciences, Karachi, Pakistan

**Keywords:** Dental implants, Ho: YAG laser, Indocyanine green, Low-level laser therapy, Repair bond strength, Non-thermal plasma, Surface conditioning, Zirconia

## Abstract

**Objectives:**

Evaluating the impact of surface conditioners, i.e., non-thermal plasma (NTP), Ho: YAG (Holmium: yttrium aluminum garnet crystal laser and Low-level laser therapy (LLLT) using Indocyanine green (ICG) on the surface topography, surface roughness (Ra) and repair bond strength (RBS) of 3% yttria-stabilized tetragonal zirconia (3Y-TZP) implant superstructure to composite.

**Methodology:**

The present lab-based study was approved by the ethical board of King Saud University under registration # FC-91-2026. The duration of the study was one month, from 15^th^ November 2025 to 15^th^ December 2025. Sixty-four 3Y-TZP discs were sectioned from ready-made zirconia. Subsequently, samples were randomly divided into four groups. Group-1: SB, Group-2: NTP, Group-3: Ho: YAG laser and Group-4: LLLT-ICG. Ra and surface topography were evaluated using profilometry and SEM, respectively. The zirconia superstructure was repaired with a composite, and the samples were artificially aged. A universal testing machine and stereomicroscope were used to assess SBS and fracture patterns. ANOVA and post hoc Tukey tests were used to compare data (p < 0.05).

**Results:**

Group-3 (Ho: YAG laser) showed the maximum roughness value (1209.42 ± 0.097 µm) and bond strength (11.43±0.12 MPa), while Group-4 (LLLT with ICG) showed the lowest Ra (679.78±0.069µm) and bond strength (6.87±0.10 MPa).

**Conclusion:**

Holmium: yttrium aluminum garnet crystal and non-thermal plasma can serve as suitable alternatives to sandblasting, as they augment the surface roughness and repair bond strength of 3Y-TZP to the composite.

## INTRODUCTION

Zirconia ceramics, particularly 3% yttria-stabilized tetragonal zirconia (3Y-TZP), are extensively utilized in dental restorations due to their biocompatibility, superior mechanical strength, low thermal conductivity, and remarkable aesthetics.^1^ Interproximal contact loss for implant superstructures can happen at an average rate of 1.9–3.6% annually after the prosthesis is placed because of the movement of neighboring teeth.^2^ The total rate of interproximal contact loss after five years post-prosthesis delivery is 80.8%. Consequently, a post-restoration repair is necessary to fill the gap resulting from interproximal contact loss and avert food impaction.^3^ Direct resin composites can be utilized for both intraoral and extraoral repairs of these prostheses during a single appointment. Nonetheless, the weak bond strength between zirconia and resin composites often leads to debonding. Consequently, it is essential to find techniques that can enhance the surface roughness (Ra) and repair bond strength (RBS) of the composite to 3Y-TZP.^4^

Sandblasting (SB) has historically been viewed as the premier surface treatment because it effectively removes impurities from the zirconia ceramic surface while also increasing Ra, thereby improving mechanical bond strength.^5^ Nonetheless, despite its efficiency in cleaning and preparing surfaces, SB also presents several significant disadvantages. The method is highly abrasive and, if not properly managed, could damage materials or cause unwanted changes in surface measurements. Additionally, the process produces a considerable amount of dust and airborne particles, which pose health risks, such as respiratory issues, for health workers.^6^

Non-thermal plasma (NTP) has become a promising method for surface treatment, capable of altering surface chemistry without causing structural damage to zirconia. Plasmas are ionized gases composed of free radicals, ions, and electrons, capable of triggering chemical reactions that significantly alter the functional groups on a material’s surface.^7^ This method allows for the conversion of typically inert, non-reactive surfaces into highly reactive surfaces, thus improving their bonding capabilities, while maintaining the substrate’s original mechanical properties.^8^

Laser irradiation is an alternative method for enhancing Ra and boosting adhesion between ceramics and resin cement. Likewise, Ho: YAG has been the subject of considerable research.^9^ The laser, producing light in the near-infrared spectrum, is commonly utilized in medicine and dentistry. Research by Alkhudhairy and his team found that Ho:YAG laser conditioning of resin-based ceramics yielded the highest bond strength compared with other investigated groups.^10^ Likewise, Photodynamic therapy (PDT) signifies noninvasive methods that are increasingly used in different areas of dental sciences.^11^ Indocyanine green (ICG) is a fluorescent pigment belonging to the tricarbocyanine dye family. Its ability to dissolve water makes it a suitable dye for use within the body. It also exhibits outstanding antimicrobial characteristics. Previous findings indicate that photosensitizer-activated PDT is effective in improving Ra and bond scores of indirect restorations to resin cement.^12^,^13^ However, data are scarce regarding their efficacy on the surface topography, Ra, and RBS of zirconia implant superstructures when bonded to composite resin, and hence there is a need for future inquiry.

The existing inquiry was founded on the assumption that no notable change exists in the Ra of 3Y-TZP discs treated with contemporary conditioning methods, i.e., NTP, Ho: YAG and ICG-PDT, as compared to SB pretreated samples. Additionally, it was expected that the RBS of the 3Y-TZP implant superstructure-to-composite would show no significant difference when treated with contemporary protocols compared with the control. Therefore, the analysis aims to assess the effects of various conditioners on the surface topography, R_a_, and RBS of the composite used in the 3Y-TZP implant superstructure.

## METHODOLOGY

The duration of the lab-based study from King Saud University, Saudi Arabia was one month, from 15^th^ November 2025 to 15^th^ December 2025. The study adhered to the Checklist for Reporting In Vitro Studies (CRIS) guidelines.

Sixty-four 3Y-TZP discs (8 mm diameter, 2 mm thickness) (Zirconzahn, Bruneck, Italy) were sectioned from ready-made zirconia blanks. Zirconia discs were placed in auto-cure acrylic resin (Meliodent; Armonk, NY). The present lab-based study was approved by the ethical board of University under registration # FC-91-2026; dated November 1, 2025.

Polishing of the substrate was performed using a silicon carbide abrasive (Buehler Ltd) in a polishing machine (Automet 500, Buehler, Esslingen, Germany) with running water, followed by ultrasonic cleaning for 3 mins in 99% isopropanol and air-drying. All discs were arbitrarily assigned to four groups based on surface treatment methods (n=16).^14^

### Group-1: SB:

3Y-TZP discs were abraded with 110-mm Al_2_O_3_ particles (Korox, BEGO) using an SB machine (Twin-Pen Sandblaster, China). The nozzle was placed perpendicular to the surface, maintained at 120 psi, and kept 2mm away for 10 secs.^2^

### Group-2: NTP:

The 3Y-TZP samples underwent NTP treatment using a plasma generator (KinPen 09, INP Greifswald, Germany) with argon (Ar) gas. Device operating parameters were 3 kVpp, 4.80 mA and a flow rate of 5.0 slm for 1 min from a distance of 10 mm above the surface.^15^

### Group-3: Ho: YAG laser:

A Ho: YAG laser (StoneLight, Minnetonka, MN, USA) was utilized to ablate 3Y-TZP discs for 20 secs under constant cooling. The parameters include 0.5 J laser energy, 8 Hz frequency, 6 W power and 2090 nm wavelength in a pulse mode.^15^

### Group-4: PDT-ICG:

A 1 mg/mL solution of ICG (Verdye, Diagnostic Green GmBH, Aschheim, Germany) was applied to the ceramic surface for approximately 5 mins, then exposed to a diode laser (Epic 10, Biolase, Irvine, CA, USA) operating at an 808 nm wavelength with a power output of 250 mW, a power density of 0.4 w/cm^2^ and an energy density of 24 J/cm^2^ for 60 secs. Following conditioning, all discs were rinsed and air dried.^16^

### Ra assessment:

Ra was assessed utilizing profilometry (NewView 6300, Zygo Corp, Middlefield, Conn). The diamond tip of the device, operating at 0.15 mm/s under a 4 N load, moved over 0.75 mm. Each specimen had one trace documented at three distinct positions (parallel, perpendicular, and oblique). The mean Ra in microns was calculated as an average of all three measurements (n= 5).^17^

### Repairing the zirconia superstructure using a composite:

The zirconia discs underwent silanization with Bis-Si (Bisco, USA). Subsequently, ALL-Bond Universal (Bisco, USA) was applied to the zirconia surface. Composite resin in shade A2 (GC, Japan) was then applied to the discs using a 4 mm diameter, 2-mm thick Tygon tube. Photopolymerization was conducted with the Valo light curing unit (Ultradent, USA) (n= 11).^1^

### Thermocycling of samples:

All bonded discs were subsequently submerged in distilled water at 37°C for 24 hours and subjected to thermocycling (Neslab Instruments Inc., USA) between 5 and 55°C for a total of 1000 cycles. Each bath immersion lasted 30 seconds and the transfer duration was 5 seconds.

### RBS testing:

A universal testing machine (Instron, England) was used to conduct the bond strength test at a crosshead speed of 1-mm/minute. Every specimen bonded interface was placed perpendicular to the jig and force was recorded till the time debonding occurred. The values of SBS were determined in Megapascals (MPa).^18^

### Assessment of bond fracture:

Debonded discs were examined using a stereomicroscope (Clinidock, Guangdong, China) at a 40× magnification to identify the type of bond failure. The bond failures were categorized into three groups: cohesive, adhesive and admixed.

### Statistical analysis:

Data were analyzed using SPSS (SPSS Inc., Chicago, IL). The Kolmogorov–Smirnov test indicated that the data adhered to a normal distribution (P > 0.05). *ANOVA*, with subsequent multiple comparisons conducted via a *post hoc Tukey test*, was used to compare data (p<0.05).

## RESULTS

### R_a_ analysis:

Table-I presents the R_a_ values of 3Y-TZP discs after different pretreatment methods. Group-3 (Ho: YAG laser) demonstrated the highest R_a_ value (1209.42 ± 0.097 µm), while Group-4 ICG-PDT showed the lowest R_a_ (679.78 ± 0.69 µm). A comparison among the groups revealed that Group-2 (NTP) (1051.54 ± 0.87 µm) and Group-3 (1209.42 ± 0.97) had comparable R_a_ values (p>0.05). R_a_ of group 4 (SB) was found to be significantly higher than the ICG-PDT group but significantly lower than samples treated with Ho:Yag and NTP group (p<0.05). In contrast, Group-4 (ICG-PDT) showed a significantly lower R_a_ than all other tested groups (p<0.05).

**Table-I:** Ra of 3Y-TZP after using various conditioning protocols.

Experimental groups		Mean ± (SD) R_a_ (µm)	p-value
Group-1	SB	903.54 ± 0.84 ^b^	p<0.05
Group-2	NTP	1051.54 ± 0.87 ^a^	
Group-3	Ho: YAG laser	1209.42 ± 0.97 ^a^	
Group-4	ICG-PDT	679.78 ± 0.69 ^c^	

! ANOVA Sandblasting (SB), Holmium: yttrium aluminum garnet crystal (Ho: YAG) laser, Non-thermal plasma (NTP), Photodynamic Therapy (PDT), Indocyanine green (ICG). Different superscript lower-case alphabets denote statistically significant differences within the same column (p<0.05).

### RBS analysis:

Table-II illustrates the RBS between composite and 3Y-TZP following various pretreatment protocols. Group-3 (Ho: YAG laser) exhibited the highest bond strength (11.43±0.12 MPa), while Group-4 (ICG-PDT) showed the lowest bond strength (6.87±0.10 MPa). Upon comparison among them, it was witnessed that Group-2 (NTP) (10.99±0.21 MPa) and Group-3 showed statistically similar results (p > 0.05). In contrast, Group-1 (SB) (8.22±0.11 MPa) recorded lower RBS values than Groups 2 and 3 but higher than Group-4 (p < 0.05).

**Table-II:** RBS of zirconia bonded to composite after using different pretreatment regimes.

Experimental groups		Mean ± SD (MPa)	p-value
Group-1	SB	8.22±0.11 ^b^	p<0.05
Group-2	NTP	10.99±0.21 ^a^	
Group-3	Ho: YAG laser	11.43±0.12 ^a^	
Group-4	ICG-PDT	6.87±0.10 ^c^	

!ANOVA Sandblasting (SB), Holmium: yttrium aluminum garnet crystal (Ho: YAG) laser, Non-thermal plasma (NTP), Photodynamic Therapy (PDT), Indocyanine green (ICG). Different superscript lower-case alphabets denote statistically significant differences within the same column (p<0.05).

### Type of fracture assessment:

Table-III presents the percentage of different fracture types among the examined groups. Samples exposed to NTP and Ho: YAG laser exhibited cohesive failure patterns most frequently. Conversely, discs exposed to SB etching exhibited mixed failures. While ICG-PDT primarily led to an adhesive failure pattern.

**Table-III:** Fracture pattern analysis among each experimental group.

Experimental groups	Group-1	Group-2	Group-3	Group-4
Adhesive	30%	20%	10%	20%
Cohesive	20%	60%	70%	10%
Admixed	50%	20%	20%	70%

## DISCUSSION

The existing inquiry was founded on the assumption that there would be no notable difference in the Ra of 3Y-TZP discs treated with contemporary conditioning methods, i.e., NTP, Ho: YAG and ICG-PDT, as compared to SB pretreated samples. Additionally, it was expected that the RBS of the 3Y-TZP implant superstructure-to-composite would show no significant difference when treated with contemporary protocols compared with the control. The results of the present study indicated that both hypotheses were entirely dismissed, as all experimental groups showed notably different Ra and bond strength compared with the SB-treated groups.

Zirconia restorations require surface pretreatment of their intaglio side to ensure long-term durability. Ho: YAG laser- and NTP-treated zirconia discs showed higher R_a_ values and bond strength among the examined groups. The highest scores in Ho: YAG laser- zirconia conditioned discs can be interpreted based on research carried out by Albakri and associates.^19^ They discovered that the Ho: YAG laser displays characteristics of both the CO_2_ and Nd: YAG lasers. A laboratory analysis conducted by Martin and colleagues showed that the Nd: YAG laser can produce zirconia R_a_ similar to that created by SB without negatively impacting the zirconia microstructure.^20^ Nd: YAG laser improves the R_a_ of zirconia by promoting the creation of surface cracks and molten areas.^20^ Moreover, CO_2_ laser exposure also leads to an increase in R_a_ by causing thermomechanical melting. Melting causes the surface of zirconia to expand, while solidification leads to rapid contraction. The combination of expansion and contraction leads to a highly rough zirconia surface.^21^ The authors believed that the combined properties of both lasers shown by the Ho: YAG laser could explain its effective performance.^20^,^21^ Likewise, the gas generated by the plasma apparatus on 3Y-TZP zirconia promotes the formation of active peroxide radicals (R-O-O), which introduce additional functional groups (C-O, C-OH) to the outer surface, thereby initiating chemical changes even on inert substances.^15^ Additionally, the NTP application possesses a cleaning capability by lowering the adsorbed hydrocarbon levels through the cleavage of C-H and C-C bonds, which ultimately enhances the bond integrity outcomes.^10^

Concerning 3Y-TZP conditioned with SB, it was observed that it led to reduced R_a_ and RBS compared to the NTP and Ho YAG laser groups but was still notably superior to the ICG-PDT pretreated group. Previous studies have reported that surfaces treated with SB not only enhance Ra and surface area by propelling high-speed abrasive particles at the material, which mechanically carve tiny peaks and valleys into the surface.^22^ This procedure eliminates undesired layers such as rust or coatings while also forming a textured surface profile, thereby improving surface wetting and enhancing mechanical interlocking when adhesive is applied.^23^ Additionally, another reason for enhanced bond strength could be that SB encourages phase alterations from the metastable tetragonal phase to the monoclinic phase.^24^ Nonetheless, it is important to remember that gentle SB can bring about favorable outcomes. While intense SB could harm the material beyond the few surface alterations and weaken the zirconia-based material rather than reinforce it.

ICG-activated PDT produced markedly reduced Ra and bond integrity compared to the other groups tested. Most recent research have focused on methylene blue PS, while only a limited number have investigated ICG PS effect on the conditioning of ceramics. ICG PS operates within the 700–900 nm wavelength range, maintaining an equilibrium between monomeric and oligomeric forms. The authors’ expectations can clarify the results, since ICG is an amphiphilic photosensitizer that absorbs water, thereby diminishing the RBS effects.^13^,^25^

Regarding failure mode, NTP- and Ho YAG laser-pretreated zirconia discs exhibited the most cohesive fractures. Whereas SB-conditioned samples exhibited predominantly admixed failure. On the other hand, ICG-PDT displayed adhesive fractures most frequently. The observed failures further support the bond strength results. Cohesive failure occurs when the adhesive fractures within its bulk, often due to inadequate surface preparation or uneven stress distribution. In contrast, adhesive failure occurs when the adhesive detaches from the substrate, commonly due to surface contamination or low surface energy.

### Clinical Innovation and Application:

This study introduces non-thermal plasma and Ho:YAG laser as innovative, non-contact alternatives to sandblasting for zirconia implant repair—addressing a critical gap in implant prosthodontics, where fractured superstructures currently necessitate complete replacement.

### Strength of the study:

For the first time, ICG-PDT activation is systematically evaluated as a surface conditioning method for ceramic biomaterials, expanding photodynamic therapy applications beyond antimicrobial and tissue ablation into materials surface engineering. The demonstration that plasma and laser treatments achieve bond strengths exceeding those of sandblasting, without the risk of alumina particle embedding—a known risk factor for peri-implantitis and ceramic degradation—provides clinicians with evidence-based, safer repair protocols that could reduce implant failure rates and healthcare costs.

### Limitations:

It should also be acknowledged that this study has certain limitations, particularly in replicating variations in the oral environment and the clinical loading forces exerted on restorations. A more comprehensive evaluation of the composite–zirconia interface using SEM would have provided deeper insights. Moreover, the exclusive use of a single PS and laser parameter may have influenced the findings. Therefore, more studies are required to confirm these outcomes.

## CONCLUSION

Holmium: yttrium aluminum garnet crystal and non-thermal plasma can be used as a suitable alternative to sandblasting, as they augment the surface roughness and repair bond strength of 3Y-TZP to composite.

### Future study direction:

Future research should explore a wider range of ICG concentrations, exposure durations, and laser parameters to determine whether optimized PDT protocols can achieve bond strengths comparable to those achieved with laser and plasma conditioning. Long-term in vivo clinical studies are warranted to assess the durability of NTP- and Ho: YAG-treated zirconia implant superstructures under functional masticatory loading and real-world oral environmental conditions.

### Author’s Contribution:

**AA:** Literature search, Data collection, study design, and conceptualization.

**MP, HA, and MN:** Manuscript writing, data analysis, and final manuscript approval.

**AA:** Is responsible and accountable for the accuracy and integrity of the present work.

## References

[1] Alkhudhairy F, Shono NN (2025). An investigation of Y-TZP surface characteristics and adhesive properties following treatment with Tri-biochemical Silica Coating, Femtosecond Laser and Nano-hydroxyapatite: A scanning electron microscopy evaluation. Pak J Med Sci.

[2] Alrabiah MA, Alkhudhairy F (2026). Effect of Sandblasting, Tribochemical Silica Coating, CO2 Laser and Plasma-Enhanced Chemical Vapor Deposition on Surface Characteristics and Shear Bond Strength of 3Y-TZP Zirconia. Cryst.

[3] Gasser TJW, Papageorgiou SN, Eliades T, Hämmerle CHF, Thoma DS (2022). Interproximal contact loss at implant sites: a retrospective clinical study with a 10-year follow-up. Clin Oral Implants Res.

[4] Falahchai M, Neshandar Asli H, Faghani M, Hendi A (2024). Effect of different surface treatments on shear bond strength of zirconia with various yttria contents. BMC Oral Health.

[5] Valverde GB, Coelho PG, Janal MN, Lorenzoni FC, Carvalho RM, Thompson VP (2013). Surface characterisation and bonding of Y-TZP following non-thermal plasma treatment. J Dent.

[6] Aljamhan A, Alkhudhairy F (2025). Impact of Hydrofluoric Acid, Ytterbium Fiber Lasers and Hydroxyapatite Nanoparticles on Surface Roughness and Bonding Strength of Resin Cement with Different Viscosities to Lithium Disilicate Glass Ceramic: SEM and EDX Analysis. Cryst.

[7] Vechiato Filho AJ, Dos Santos DM, Goiato MC, De Medeiros RA, Moreno A, Bonatto LDR (2014). Surface characterization of lithium disilicate ceramic after nonthermal plasma treatment. J Prosthet Dent.

[8] Silva NRFA, Coelho PG, Valverde GB, Becker K, Ihrke R, Quade A (2011). Surface characterization of Ti and Y-TZP following non-thermal plasma exposure. J Biomed Mater Res - Part B Appl Biomater.

[9] Shono NN, Alkhudhairy F (2025). Repair Bond Strength in Hybrid Ceramic Preconditioned With Ho: YAG Laser and Toluidine Blue, Activated by Low-Level Laser Therapy, Using Composites With Varying Viscosities: An Analysis of Surface Topography and Elemental Analysis. Microsc Res Tech.

[10] Alkhudhairy F, AlFawaz YF (2024). Pretreatment of Hybrid Ceramics Using Ho: YAG, Low-Level Laser Therapy Activated Malachite Green and Non-Thermal Plasma on Surface Roughness, Bond Strength and Color Change, SEM and EDX Analysis. Ceramics.

[11] Alanazi AM, Khan AA, Mahmood A, Kamal MA, Baig EA (2024). The antibacterial efficiency of Chitosan photodynamically activated Phycocyanin and Morinda Oleifera against S.mutans and the bonding strength between composite resin and caries-affected dentin. Pak J Med Sci.

[12] Alrefeai MH, Aljamhan AS, Alhabdan A, Alzehiri MH, Naseem M, Alkhudhairy F (2022). Influence of methylene blue, Riboflavin and indocyanine green on the bond strength of caries-affected dentin when bonded to resin-modified glass ionomer cement. Photodiagnosis Photodyn Ther.

[13] Kumari U, Zafar T, Shafqat S, Askary SH, Qasim M, Kamran MA (2023). Caries-affected dentin disinfection using Triphala, Indocyanine green and Potassium Titanyl Phosphate laser and their effect on adhesive bond strength. Photodiagnosis Photodyn Ther.

[14] Henriques B, Fabris D, Souza JCM, Silva FS, Carvalho Ó, Fredel MC (2018). Bond strength enhancement of zirconia-porcelain interfaces via Nd: YAG laser surface structuring. J Mech Behav Biomed Mater.

[15] Liu J, Tu S, Wang M, Chen D, Chen C, Xie H (2025). The influence of different factors on the bond strength of lithium disilicate-reinforced glass–ceramics to Resin: a machine learning analysis. BMC Oral Health.

[16] Alkhudhairy F, Naseem M, Ahmad ZH, Alnooh AN, Vohra F (2019). Efficacy of phototherapy with different conventional surface treatments on adhesive quality of lithium disilicate ceramics. Photodiagnosis Photodyn Ther.

[17] Maawadh AM, Almohareb T, Al-Hamdan RS, Al Deeb M, Naseem M, Alhenaki AM (2020). Repair strength and surface topography of lithium disilicate and hybrid resin ceramics with LLLT and photodynamic therapy in comparison to hydrofluoric acid. J Appl Biomater Funct Mater.

[18] Alsunbul H, Almutairi B, Aljanakh M, Abduljabbar T (2023). Hybrid ceramic repair strength, surface roughness, and bond failure, using methylene blue-activated low-level laser therapy, Carbon dioxide, and Ti: Al2O3 laser. Photodiagnosis Photodyn Ther.

[19] Albakri AS (2024). Pretreatment of Hybrid Ceramics Using Alumina (Al 2 O 3 ) Nanoparticles, Hydrofluoric Acid and Holmium: YAG Laser for Optimizing Surface Roughness, Shear Bond Strength and Topography. J Biomater Tissue Eng.

[20] Martins FV, Mattos CT, Cordeiro WJB, Fonseca EM (2019). Evaluation of zirconia surface roughness after aluminum oxide airborne-particle abrasion and the erbium-YAG, neodymium-doped YAG, or CO2 lasers: A systematic review and meta-analysis. J Prosthet Dent.

[21] Rocca JP, Fornaini C, Brulat-Bouchard N, Bassel Seif S, Darque-Ceretti E (2014). CO2 and Nd: YAP laser interaction with lithium disilicate and Zirconia dental ceramics: A preliminary study. Opt Laser Technol.

[22] Kim HK, Ahn B (2021). Effect of Al_2_O_3_ sandblasting particle size on the surface topography and residual compressive stresses of three different dental zirconia grades. Materials (Basel).

[23] Su N, Yue L, Liao Y, Liu W, Zhang H, Li X (2015). The effect of various sandblasting conditions on surface changes of dental zirconia and shear bond strength between zirconia core and indirect composite resin. J Adv Prosthodont.

[24] Finger C, Stiesch M, Eisenburger M, Breidenstein B, Busemann S, Greuling A (2020). Effect of sandblasting on the surface roughness and residual stress of 3Y-TZP (zirconia). SN Appl Sci.

[25] Alshahrani A, Abrar E, Maawadh AM, Al-Hamdan RS, Almohareb T, AlFawaz Y (2020). Management of caries affected dentin (CAD) with resin modified glass ionomer cement (RMGIC) in the presence of different caries disinfectants and photosensitizers. Photodiagnosis Photodyn Ther.

